# Clinical and functional outcomes of the silo technique in the management of diabetic calcaneal osteomyelitis

**DOI:** 10.1186/s40842-025-00238-4

**Published:** 2025-11-03

**Authors:** Nurarif Nurhashim, Ken Meng Tai, Abdul-Hadi Kafagi, Anand Pillai

**Affiliations:** 1 Faculty of Biology, Medicine and Health, The University of Manchester, Manchester, UK; 2 Department of Orthopaedics, Wythenshawe Hospital, Manchester University Hospitals NHS Foundation Trust, Manchester, UK

**Keywords:** Ambulatory status, Calcaneal osteomyelitis, Calcaneus, Calcium sulphate, Cerament, Diabetic ulcer, Heel ulcer, Hydroxyapatite, Limb salvage, Partial calcanectomy

## Abstract

**Background:**

Partial calcanectomy is an established alternative to amputation in diabetic calcaneal osteomyelitis, with recent studies utilising adjuvant local antibiotic delivery devices to improve outcomes. The Silo technique is a novel approach involving an antibiotic-loaded hydroxyapatite calcium sulphate bioceramic (Cerament G or V) implanted into pre-drilled holes in the calcaneus.

**Method:**

This retrospective case series involved 30 patients with chronic diabetic calcaneal osteomyelitis that underwent partial calcanectomy with Cerament G or V application via the Silo technique between 2014 and 2024. Patients were further followed up on their ambulatory status via telephone consultation. Primary outcomes were infection eradication, ulcer healing, limb salvage, patient mortality and ambulatory status.

**Results:**

Infection eradication was achieved in 29 (97%) patients, ulcer healing in 27 (90%), and limb salvage in 28 (93.3%). Ulcer recurrence occurred in 8 (26.7%) patients. The all-cause mortality rate was 6.7% at 1-year and 43.3% at 5-years. With regards to ambulatory status, 6 (20.0%) patients improved their ambulatory status from baseline, 20 (66.7%) maintained their baseline ambulatory status, and 4 (13.3%) deteriorated in ambulatory status from baseline.

**Conclusion:**

The Silo technique for diabetic calcaneal osteomyelitis demonstrates promising clinical outcomes, including infection eradication, ulcer healing, ulcer recurrence, limb salvage, 1-year mortality, and mobility. Further prospective studies with larger cohorts and randomised controlled trials are warranted to validate these exploratory findings and to better understand the factors influencing both short and long-term outcomes.

**Supplementary Information:**

The online version contains supplementary material available at 10.1186/s40842-025-00238-4.

## Background

Diabetes is estimated to affect 9.3% of the global population, and is projected to reach 10.2% by 2030 [1]. Diabetic foot ulcers (DFU) are a common complication of diabetes, with diabetic patients having a 25% lifetime risk of such ulcers [2]. Among these, the rates of infection for DFUs are approximately 60% [3]. DFUs involving the heel are one of the most common causes for calcaneal osteomyelitis and are associated with extended healing time and poorer prognosis compared to ulcers involving other locations on the foot [4–6].

The combination of factors caused by hyperglycaemia including peripheral neuropathy, an impaired immune response, and peripheral vascular disease, negatively affects wound healing in diabetic osteomyelitis, which predisposes the development of chronic osteomyelitis (COM) [2, 4]. COM is characterised by the histopathological findings of bone destruction and the development of necrotic bone due to the progressive inflammation [7]. When wound closure or ulcer healing fails, below knee amputation (BKA) becomes a definitive management option for high-risk diabetic patients with heel ulceration. Diabetics overall have a five times greater risk for BKA compared to non-diabetics [8].

In order to reduce the risk of BKA, several strategies have been described to achieve limb salvage in cases of calcaneal osteomyelitis. Among them, partial calcanectomy (PC) via split heel approach was first described in literature by Gaenslen as an alternative treatment for calcaneal COM with the potential for limb salvage [9]. Recently, PC has become an established alternative to BKA in high-risk patients with DFUs, with several studies reporting good outcomes for ulcer healing, limb salvage and post-operative mobilisation [10–14]. A systematic review of 76 patients undergoing PC found it to be a viable alternative to BKA in osteomyelitis, preserving functional use when supported with orthotics [8]. Alongside PC to resect infected bone, the management of diabetic calcaneal osteomyelitis generally involves surgical debridement to completely remove infected surrounding soft tissue. This is followed by long term systemic antibiotics based on culture results, regular wound care, and follow up in clinic to monitor for infection recurrence.

Alongside PC, there has been increased use of adjuvant synthetic materials loaded with antibiotics to deliver antibiotics locally to treat osteomyelitis [15–19]. This not only fills the dead space left from debridement and resection but also has the advantage of delivering antibiotics to the bone at higher concentrations not achievable with systemic antibiotics. They allow antibiotics to be delivered at concentrations over 100 times the minimum inhibitory concentration to sensitive microorganisms, reducing the risk of infection recurrence, with potential effectiveness against antibiotic-resistant microorganisms commonly found in biofilms that develop from bone infections [20, 21].

The use of PC combined with the administration of local antibiotics has been shown to improve patient outcomes in treating calcaneal osteomyelitis [22, 23]. Although, one study found no benefit to the application of local antibiotics [18]. Among these antibiotic delivery systems, a combination of hydroxyapatite and calcium sulphate biocomposite loaded with antibiotics has shown efficacy at clearing infection in osteomyelitis and for managing bone void [15, 22–25].

There have been several methods described to apply the various forms of local antibiotic delivery devices to treat COM, such as using beads or impregnated collagen sponges [26]. One method described by Drampalos et al. as the Silo technique involves drilling multiple silo-like tunnels are into the calcaneus and applying a gentamicin-loaded calcium sulphate and hydroxyapatite bioceramic (Cerament G). This approach reported successful infection eradication and ulcer healing in all 12 patients. Although most applications of local antibiotic delivery systems are also used as dead space management, PC does not always result in any dead space. However, the Silo technique involves creating bone void by drilling tunnels to inject the bioceramic [27].

Although the outcomes reported from the Silo technique are based on a relatively small sample size, this novel approach of implanting a widely used and effective adjuvant local antibiotic delivery has demonstrated excellent outcomes. However, the previous study is currently the only literature describing the use of the Silo technique. The small sample size provides only a limited indication of outcomes. There are currently no other studies reporting on the use of this technique and its outcomes, particularly how it affects long term functional outcomes. Hence, this retrospective case series aims to report the clinical and functional outcomes of patients undergoing PC with application of antibiotic-loaded hydroxyapatite ceramic (Cerament) via the Silo technique.

## Materials and methods

### Study design and population

**Fig. 1:**
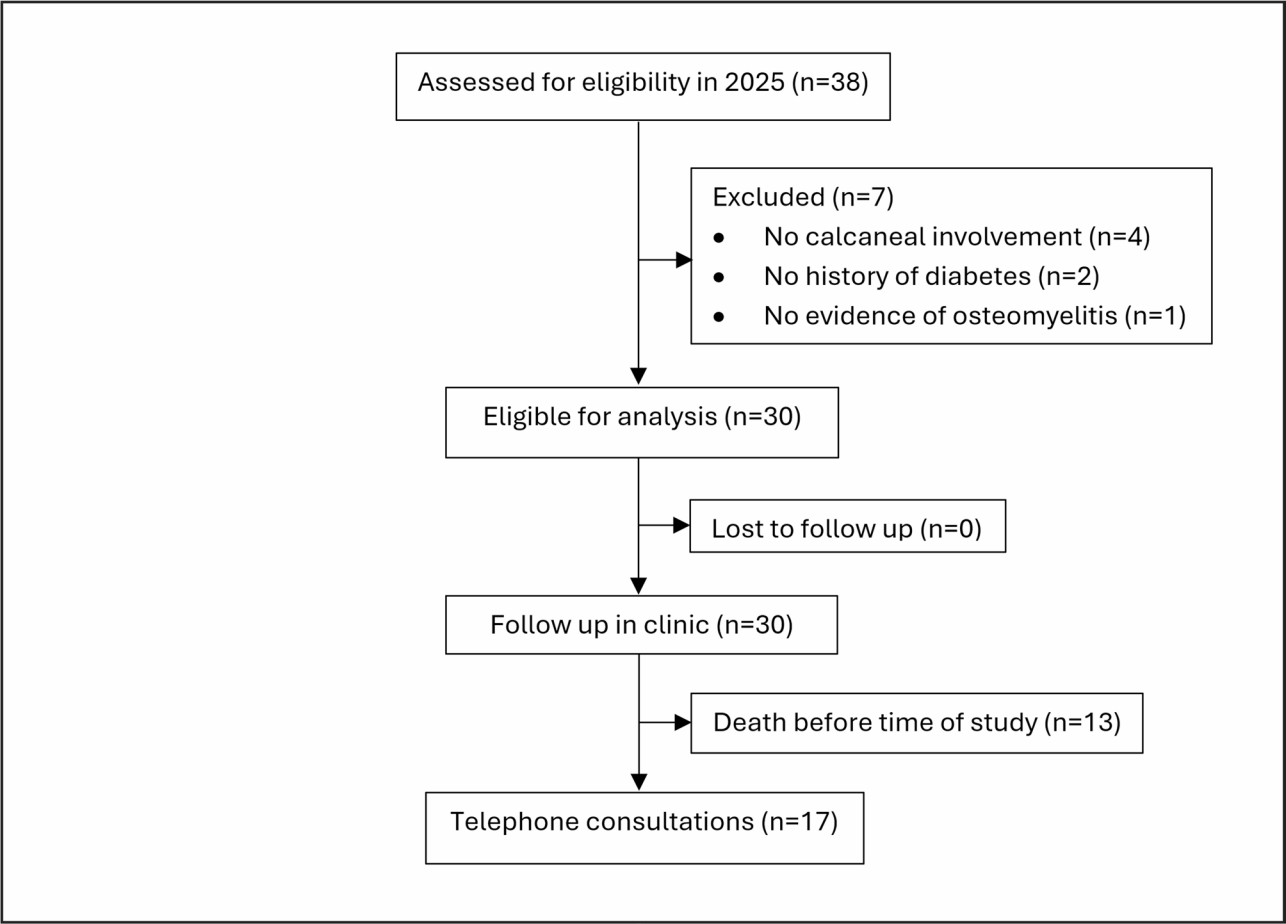
Patient flow diagram summarising patient eligibility and follow-up

This is a retrospective single centre study undertaken at a teaching hospital in the northwest of England. Patients admitted to Wythenshawe Hospital, Manchester University NHS Trust who underwent a PC with local antibiotic Cerament implementation via the Silo technique from 2014 to 2024 were identified. All patients in this study had chronic osteomyelitis (COM), which was defined for the purposes of this study as symptoms persisting for at least six months, confirmed by magnetic resonance imaging (MRI) scans, supported by clinical presentation and microbiological evidence. Further bone scans were carried out to confirm the presence of osteomyelitis in instances of diagnostic uncertainty. Inclusion criteria included patients aged 18 years and above with chronic diabetic osteomyelitis involving the calcaneus who underwent PC with the Silo technique to implant a 40% hydroxyapatite 60% calcium sulphate biocomposite loaded with either Gentamicin or Vancomycin (Cerament G or V). Exclusion criteria included patients without diabetes or chronic osteomyelitis, those with soft tissue infections without bone involvement and cases of chronic osteomyelitis without calcaneal involvement. Patients were treated between 2014 and 2024 and were assessed for eligibility for the study and contacted for telephone follow up in 2025. The patient selection and inclusion process are summarised in Fig. 1.

### Pre-operation details

Patient records were accessed electronically to obtain information on patient demographics, comorbidities, investigation results, radiographs, operation notes and clinic notes. Relevant investigation results were collected including pre-operative C-reactive protein (CRP), glycated haemoglobin (HbA1c), and cultures and sensitivities from wound and bone samples. Patient demographic data included age, sex, smoking status, and body mass index (BMI). Pre-operative HbA1c results measured in mmol/mol were used to classify patients based on their diabetic control. Patients with 42 to 58 mmol/mol were categorised as good diabetic control, 59 to 75 mmol/mol as suboptimal diabetic control, and more than 75 mmol/mol as poor diabetic control. BMI classifications were adapted based on the National Institute for Health and Care Excellence (NICE) guidelines, with a BMI of less than 18.5 classified as underweight, normal as 18.5 to 24.9, overweight as 25.0 to 29.9, obese as 30 to 39.9, and severely obese as over 40 [28]. Patient comorbidities included diabetes, chronic kidney disease (CKD), Charcot arthropathy and peripheral arterial disease (PAD).

CKD was defined as an estimated glomerular filtration rate (eGFR) of less than 60mL/min/1.73m^2^. PAD was defined as an ankle brachial pressure index of less than 0.9 and was confirmed on admission via Duplex ultrasound. Based on comorbidities and physiological status, all patients were classed as Cierny-Mader (C-M) class B^LS^ hosts (local and systemic compromise), although this was not part of the inclusion criteria [29]. MRI scans of the foot were performed for pre-operative planning and used to determine suitability for the Silo technique based on the extent of the wound. The presence of sepsis and the general health of the patient was also considered.

### Operative details and interventional description

The operative technique for PC and Cerament implantation is adapted from Drampalos, described as the Silo technique, which took place under the same centre as this study [27]. A posterior longitudinal incision is made onto the inferior surface of the heel to the ulcer or via splitting the Achilles tendon. All infected and non-viable bone is debrided, with multiple samples of bone and deep tissue sent for cultures and sensitivities. Four to five Silo-like tunnels are drilled into the calcaneus towards the posterior subtalar joint using a 3.2 mm drill bit via X-ray guidance. The wound is then irrigated and dried, followed by injection of the Silo tunnels with Cerament. The wound is then closed either primarily in theatre or via vacuum-assisted closure. The decision to resect the Achilles tendon insertion during PC was decided based on intraoperative assessment of the extent of osteomyelitis on the calcaneus and the involvement of the Achilles tendon insertion.

### Postoperative and follow-up details

Patients were managed and followed up for a minimum of one year by a multi-disciplinary team (MDT) of orthopaedic, plastic, and vascular surgeons, microbiologists, diabetic medicine specialists, physiotherapists, podiatrists and diabetes specialist nurses.

All patients were initially given systemic antibiotics which were stopped at least 2 weeks pre-operatively, guided by cultures if available. Intravenous gentamicin and teicoplanin were given intra-operatively. All patients were then commenced on systemic antibiotics for a minimum of 2 weeks. The selection and duration of antibiotics did not follow a standardised protocol and was decided by the MDT based on clinical response, infective markers, and cultures and sensitivity results.

After discharge all patients were followed up in clinic every two weeks for the first month then monthly by the foot and ankle team. Once wounds were fully healed, patients were then followed up by the podiatry team with assistance from diabetes specialist nurses and physiotherapists.

All patients were non-weight bearing (NWB) after surgery, with a period decided by the MDT based on assessment in hospital. In some cases, this was followed by off-loading with a cast or a boot with support from physiotherapy and podiatry. Physiotherapy assessment notes and clinic notes were reviewed to determine baseline ambulatory status and post-operative ambulatory status at 6 months or more after the index procedure. Telephone consultations were conducted at the time of the study to determine long-term functional outcomes. A questionnaire-based script was used where each patient was asked about their current ambulatory status, and whether they required any mobility aids. Patients were specifically asked whether they were able to ambulate indoors and outdoors. Patients that were bedbound or wheelchair bound were asked whether they were able to transfer independently or if they required a hoist to transfer.

### Study endpoints and data collection

All patients were followed up in clinic to assess ulcer healing and monitor for infection recurrence or complications. Based on the International Working Group on the Diabetic Foot (IWGDF) guidelines, ulcers were defined as healed if there was full re-epithelisation of the wound without drainage, while ulcer recurrence was defined as a previously healed ulcer developing a new full thickness ulcer at the same site of a previously healed wound [30]. Infection eradication was defined as clinical and radiological improvement with the resolution of infective markers. Clinical improvement includes the absence of clinical signs of infection, such as induration, swelling, erythema. Radiological changes include the absence of soft tissue swelling, good uptake of Cerament in the calcaneus with the absence of loosening or new lucency of the bone. Infective markers include white cell count, CRP and erythrocyte sedimentation rate.

Primary outcomes were infection eradication, ulcer healing, limb salvage, patient mortality from all causes and ambulatory status. Secondary outcomes were culture results, duration of post-operative antibiotics, and percent of calcaneus resected.

Ambulatory status was categorised into ambulatory outdoors without aids, ambulatory outdoors with aids, ambulatory indoors with aids, ambulatory for transfers only, and non-ambulatory. Mobility aids included walking sticks, Zimmer frames, and crutches. Ambulatory outdoors without aids included patients that could ambulate outdoors and indoors without aids. Ambulatory outdoors with aids included patients that could ambulate outdoors and indoors with aids. Ambulatory indoors with aids included patients that could only ambulate indoors with aids. Ambulatory for transfers only included patients that could only ambulate independently for transfers. Non-ambulatory included patients that were wheelchair bound or bedbound requiring the use of a hoist for transfers. Change in ambulatory status was based on their pre-operatively ambulatory status and their latest recorded ambulatory status based on either physiotherapy assessments and clinic notes for deceased patients or from telephone follow up for patients that survived at the time of the study.

Pre-operative and post-operative lateral radiographs of the foot were compared to calculate the difference between the 2-dimensional surface area of the calcaneus by measuring the approximate height and length of the calcaneus in order to estimate the percent of calcaneus resected [13, 31]. These were then categorised into either < 25%, between 25 and 50%, or > 50% of calcaneus resected. The involvement of the Achilles tendon insertion was also assessed.

### Statistical analysis

Descriptive statistics were used for this study, utilising percentages and mean, median and mode as measures of central tendency, with corresponding standard deviations (SD) included. Kaplan-Meier survival curves were used to assess time-dependent outcomes, including time to infection eradication and time to healing. Statistical analysis was conducted using the Statistical Package for Social Sciences (SPSS) 30.0 software.

## Results

### Baseline characteristics

30 patients (30 limbs) met the inclusion criteria and were included in the study with a mean age of 66.8 years (range of 38 to 91 years, SD 13.8) with 17 (56.7%) males and 13 (43.3%) females. Pre-operative CRP was measured at a mean of 121.7 mg/L (range of 8.0–451.0 mg/L, SD 87.8). Mean HbA1c was 68.7mmol/mol (range of 34.0 to 111.0mmol/mol, SD 17.9). 14 (46.7%) patients had CKD at time of diagnosis. 16 (53.3%) had PVD, 9 (30.0%) had Charcot arthropathy. The average BMI was 31.4 (range of 20.6 to 43.0, SD 7.6), with 7 patients classed as obese and 5 classed as severely obese. 11 (36.7%) patients were smokers. (Table 1)

**Table 1:** Baseline characteristics of the 30 patients included in the study, represented with the number of patients or the mean with corresponding percentages and SD

Patient demographics	
Age, years, mean (SD)	66.8 (13.9)
Male, n (%)	17 (56.7)
Smoker, n (%)	11 (36.7)
BMI, kg/m^2^, mean (SD)	31.4 (7.6)
CKD, n (%)	14 (46.7)
Charcot, n (%)	9 (30.0)
PAD, n (%)	16 (53.3)

Abbreviations: CKD, chronic kidney disease; PAD, peripheral arterial disease

### Clinical outcomes

Infection was eradicated in 29 (97%) patients with a mean time to eradication of infection of 9.2 weeks (range of 4 to 24 weeks, SD 14.0). Ulcer healing occurred in 27 (90%) patients with a mean time to healing of 20.0 weeks (range of 5 to 52 weeks, SD 4.9). Ulcer recurrence occurred in 8 cases (26.7%) all of which underwent a second debridement in theatre with 4 of these undergoing a third debridement in theatre. These patients underwent their second and third debridement 6 weeks following the previous debridement.

Limb salvage was achieved in 28 (93.3%) patients. For the remaining two patients, one underwent a BKA 5 months after PC and the other underwent a Syme amputation where the heel was preserved 4 months after PC.

13 patients died within 5 years of the index procedure, representing an overall and 5-year all-cause mortality rate of 43.3%. Of these, 2 died within the first year post-operatively, representing a 1-year mortality rate of 6.7%. 7 patients died by the second year, 2 by the third year, and 1 patient by the fourth and fifth year respectively.

Between male and female patients, all the female patients demonstrated successful infection eradication and ulcer healing. In comparison, 2 male patients did not show successful ulcer healing, and one did not show successful infection eradication. All-cause mortality was higher in females compared to males at 54% and 35% respectively.

**Fig. 2:**
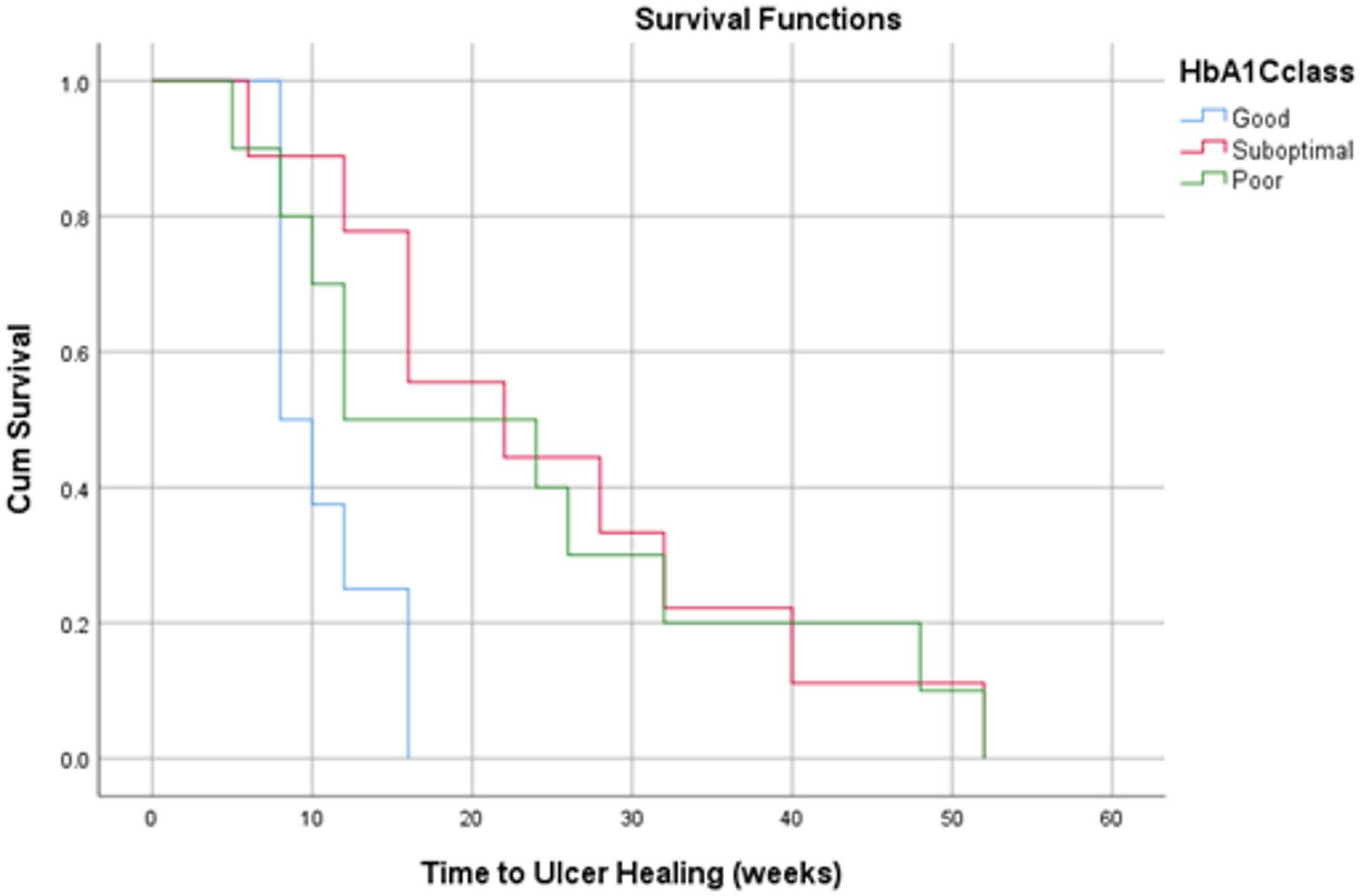
Kaplan-Meier curve demonstrating time to ulcer healing in weeks based on pre-operative HbA1c levels classified according to diabetic control

**Fig. 3:**
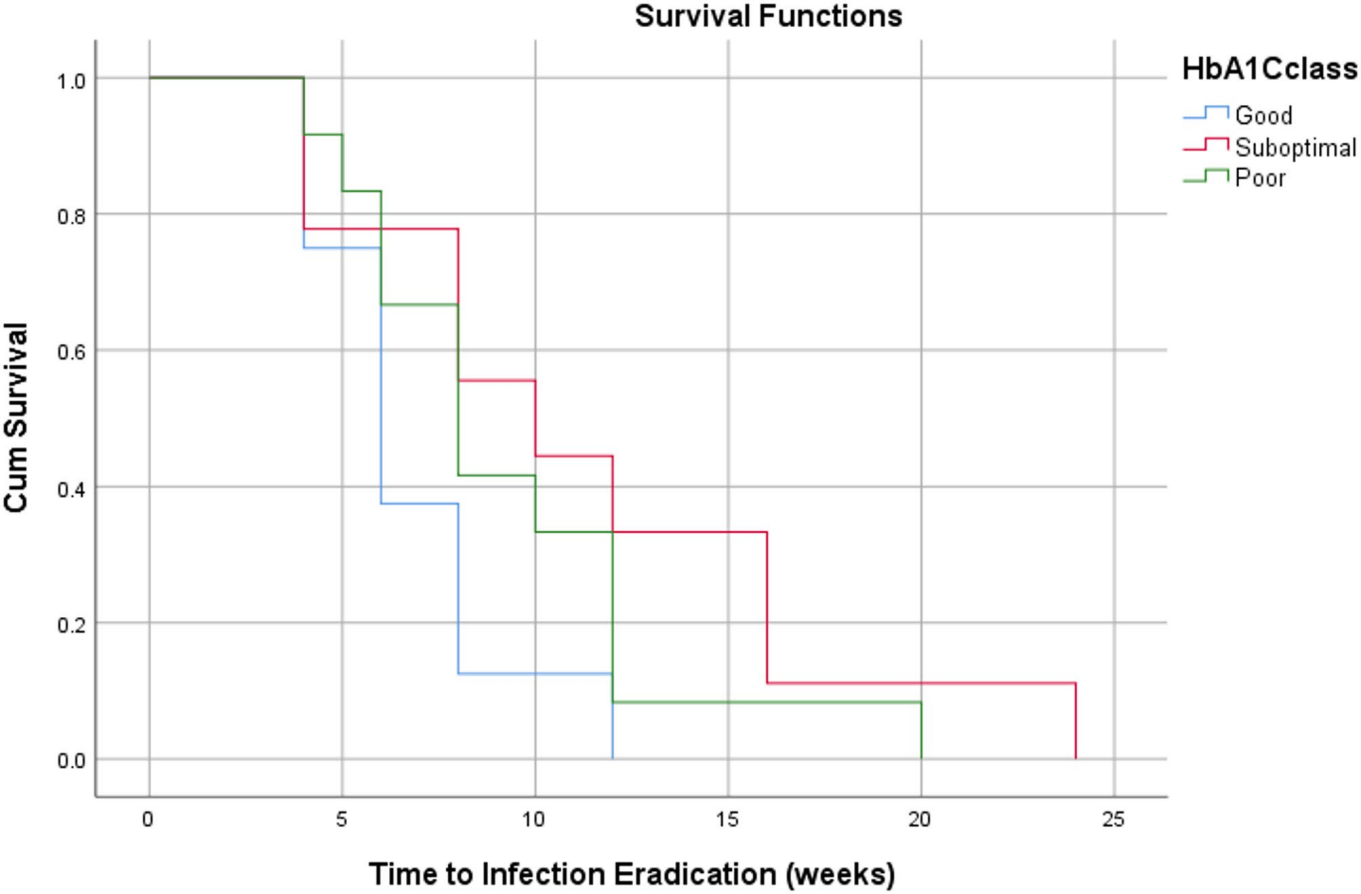
Kaplan-Meier curve demonstrating time to infection eradication in weeks based on pre-operative HbA1c levels classified according to diabetic control

**Fig. 4:**
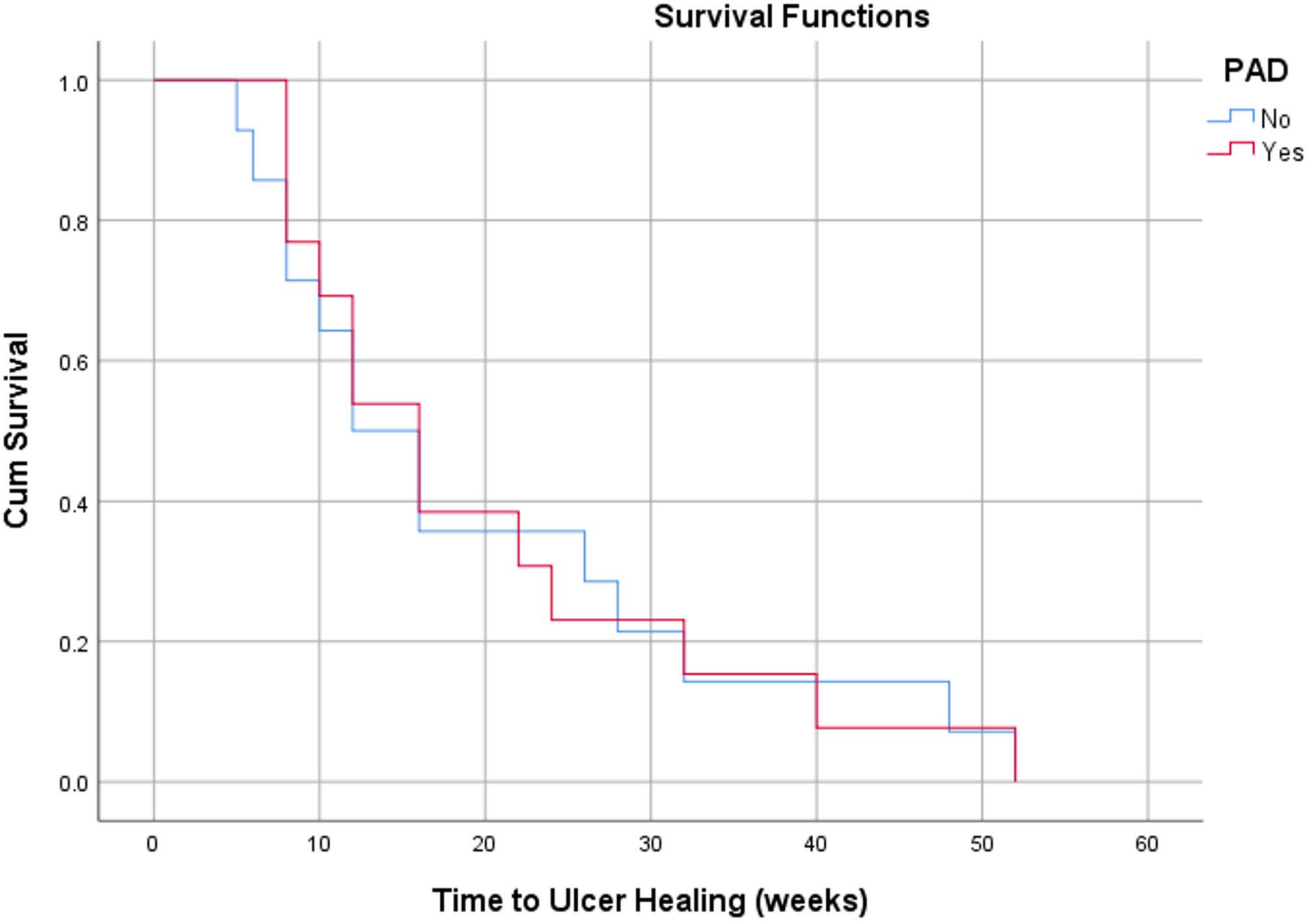
Kaplan-Meier curve demonstrating time to ulcer healing in weeks based on the presence of peripheral arterial disease (PAD)

**Fig. 5:**
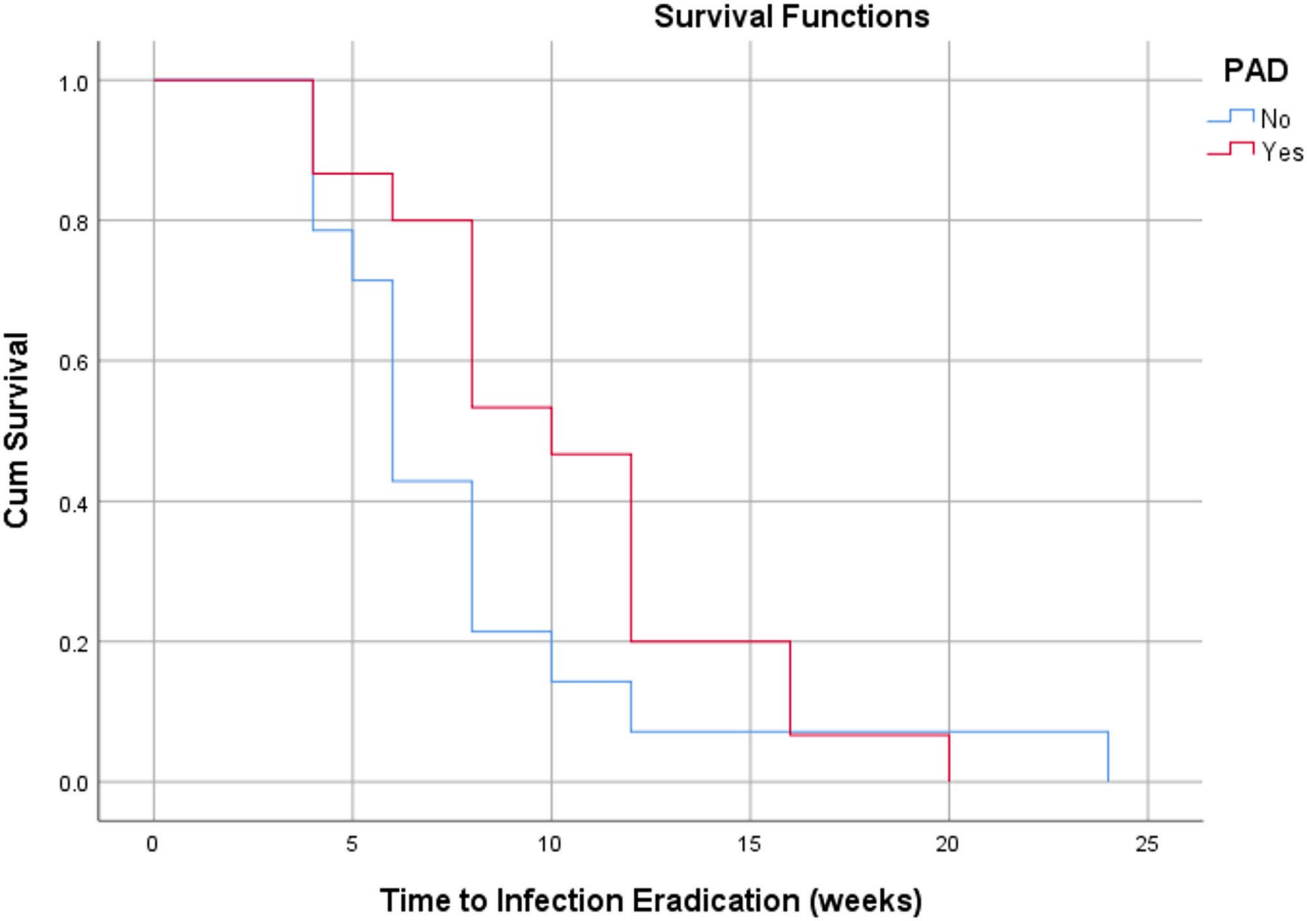
Kaplan-Meier curve demonstrating time to infection eradication in weeks based on the presence of peripheral vascular disease (PAD)

Based on survival analysis of time-dependent outcomes, patients with good diabetic control demonstrated a faster time to ulcer healing and infection eradication compared to those with suboptimal and poor diabetic control (Figs. 2 and 3). Patients with PAD did not show a significant difference in time to ulcer healing compared to those without (Fig. 4). However, patients with PAD demonstrated a faster time to infection eradication (Fig. 5).

### Functional outcomes

Follow up telephone consultations were conducted for 17 patients that were alive at the time of the study, representing a mean follow up time from index surgery of 2.4 years (range of 0.67 to 7.3 years). 6 (20.0%) patients improved their ambulatory status from their baseline, 20 (66.7%) maintained their ambulatory status from their baseline, while 4 (13.3%) patients showed a deterioration in ambulatory status from their baseline. The ambulatory status of all patients pre-operatively, 6 months or more post-operatively, and at the time of follow up are summarised in Table 2.

**Table 2:** Ambulatory status of 30 patients at baseline and ≥ 6 months post-operatively, and ambulatory status of 17 patients at follow up

Ambulatory status	Baseline (*n* = 30)	≥ 6 months post-operative (*n* = 30)	Follow up (*n* = 17)
Ambulatory without aids	**3**	**3**	**5**
Ambulatory outdoors with aids	**4**	**4**	**8**
Ambulatory indoors with aids	**16**	**15**	**2**
Ambulatory for transfers only	**4**	**6**	**0**
Non-ambulatory	**3**	**2**	**2**

### Secondary outcomes

All cases had positive culture results, with 25 (83.3%) showing polymicrobial involvement and 5 (16.7%) showing monomicrobial involvement. The most frequently isolated pathogen across all three debridements was *Staphylococcus aureus* (20, 26.0%), followed by Corynebacterium striatum (9, 12%). The frequencies of other isolated pathogens are summarised in Supplementary Material, Table S1. Across all three debridements, *Staphyloccocus aureus* remained the most frequently isolated pathogen. *Corynebacterium striatum was the second most frequently isolated pathogen from the first and second debridements.*

All patients received systemic antibiotics post-operatively, for a mean duration of 20.0 weeks (range of 5 to 52 weeks, SD 4.9). 19 patients had < 25% of calcaneus resected, 7 had between 25 and 50% of calcaneus resected, and 4 had > 50% of calcaneus resected. 17 (56.7%) had no tendoachilles involvement, while 13 (43.3%) had the tendoachilles attachment either partially or fully resected.

Cerament G was utilised in 23 patients undergoing the Silo technique, with 22 showing successful infection eradication and 20 showing successful ulcer healing. The remaining 7 patients were treated with Cerament V with all patients showing successful infection eradication and ulcer healing.

## Discussion

Chronic calcaneal diabetic osteomyelitis of the heel is difficult to treat and often leads to BKA in high-risk patients when debridement fails to eradicate the infection or wound closure is unsuccessful. This is due to the physiological status and comorbidities of these patients as well as the calcaneus serving a vital role in weight-bearing and having limited microvasculature and surrounding soft tissue which overall negatively affects healing [4, 32, 33].

This study reports a rate of infection eradication of 97%. Similar studies utilising antibiotic delivery alongside PC reported a rate of infection eradication of 75–85% [33, 34]. Another study and systematic review investigating the efficacy of local antibiotic delivery to treat osteomyelitis in general reported a rate of infection eradication of between 92 and 95.2% [16, 24]. The rate of ulcer healing was 90% with a rate of ulcer recurrence of 26.7%. This is in line with another study involving PC which found a healing rate of 77% [10]. Another study assessing the long term outcomes of PC reported a recurrence rate of 18% [6]. However, both studies only utilised PC without any local antibiotic delivery. For ulcer recurrence, a systematic review reported a 32% rate of recurrence in 202 cases of PC [23]. Limb salvage was achieved in 93.3% of patients which is consistent with other studies utilising local antibiotic delivery devices that demonstrated rates of limb salvage between 75 and 95% [15, 34]. Notably, all these studies utilising local antibiotic delivery with similar outcomes also utilised calcium sulfate, although with different methods of application. However, many of these studies are also case series with limited sample sizes and no comparative cohorts.

This study had an all-cause mortality rate of 43.3% with a 1-year all-cause mortality rate of 6.7%. Other studies report a mortality rate after PC of between 9.8 and 12% [6, 10, 35]. Although the 1-year mortality is relatively low compared to findings from similar studies, the overall 5-year mortality was relatively high. This could be attributed to several factors, including comorbidities, which many of the patients in this study present with, or due to complications following the operation itself. However, due to the lack of data on cause of mortality, this cannot be fully addressed in this study and cannot be solely attributed to undergoing PC with the Silo technique. While PC is a viable limb salvage procedure, its impact on long-term function remains a key concern due to the calcaneus’s critical role in weight-bearing. Our study contributes to the literature by reporting long-term functional outcomes following PC, offering valuable insight into whether salvaged limbs retain meaningful function years after surgery. Although mobility is commonly reported, few studies have examined it in detail [9, 14]. One such study by Oliver explored the relationship between the extent of calcaneal resection and lower limb function using the Lower Extremity Functional Scale [14].

This study recorded the ambulatory status of patients at 3 points in time, at baseline and at 6 months or more after PC for all 30 patients based on physiotherapy assessments, and at telephone follow up for the 17 patients that were alive at the time of the study. Of the three patients that were non-ambulatory before PC, two remained non-ambulatory post-operatively, while the other improved to ambulatory indoors with aids. Two of the four patients that were ambulatory for transfers became ambulatory post-operatively. However, changes in ambulatory status cannot solely be attributed to PC with the Silo technique and could have been influenced by other factors such as ageing, unrelated comorbidities, or other operations. Overall, these findings demonstrate a positive outcome for mobility after undergoing PC, with most patients either maintaining or improving their ambulatory status, with a minority deteriorating in their ambulatory status.

Ravine reported 76% of their patients remained ambulatory post-operatively, with all that were ambulatory without aids pre-operatively remaining ambulatory post-operatively, and no patients that were ambulatory pre-operatively became non-ambulatory [10]. A systematic review found that 75% of 76 patients undergoing PC maintained their ambulatory status post-operatively, with 9.2% improving ambulatory status and no patients deteriorating in their ambulatory status [8].

All patients that improved in their ambulatory status had < 25% of calcaneus resected. Conversely, of the 4 patients that had > 50% of calcaneus resected, two maintained their ambulatory status, one deteriorated in ambulatory status, and one improved in ambulatory status, although all still were ambulatory at some level post-operatively. These findings suggest that the amount of calcaneus resected may impact functional outcomes. However, Oliver reported no significant difference in ambulatory status between patients that had < 50% of calcaneus resected compared to those with > 50% resected, instead noting that the Achilles attachment had a larger impact on mobility [13]. In this study, of the 13 patients that had the Achilles attachment resected, 2 deteriorated in ambulatory status from baseline, 1 improved their ambulatory status, and the remaining 10 maintained their ambulatory status. All 3 that had a change in ambulatory status had the Achilles attachment completely resected.

Although some patients experience some reduction in mobility following PC, there is a reasonable expectation that patients achieve at least household ambulation with mobility aids after PC [8, 10]. Mobilisation after PC can be further supported with the use of orthotics to aid with weightbearing, particularly in cases where a majority of the calcaneus has been resected [13, 36]. All the patients in this study were provided a boot as needed and were arranged to have orthotics provided at discharge to support their ambulation. These were used at least until the ulcer was healed. Patients were further assessed after removal of their orthotics.

The most frequently isolated microorganism was *Staphylococcus aureus* with 13 (43.3%) cases isolating it at first debridement. This is consistent with other studies and a systematic review which reported rates of *Staphylococcus aureus* isolation of 42.7% [6, 15, 23]. Two studies reported the presence of MRSA as a factor delaying wound closure [12, 37]. This study identified 4 cases that isolated MRSA. Three patients showed successful ulcer healing at 52, 16, and 24 weeks, with the latter dying 5 years after PC. The fourth patient’s ulcer did not heal and proceeded to BKA 4 months after PC, representing one of the two cases of unsuccessful limb salvage. The remaining three cases represent a 75% rate of ulcer healing and infection eradication. Among these 3 cases, there was a mean time to healing and eradication of infection of 30.7 weeks and 11.3 weeks respectively. Both the mean time to healing and infection eradication were slower among these 3 cases compared to the non-MRSA patients that demonstrated a mean time to ulcer healing and infection eradication of 18.6 and 9.0 weeks respectively. Due to the small number of MRSA isolated and the variation of outcomes, further research would be needed to investigate the effect of MRSA on ulcer healing.

As a retrospective case series from a single centre with a sample size of 30 patients, this study is primarily descriptive in nature and not designed to establish causality or definitive predictors of outcomes. While the absence of a control group limits comparative analysis to control for confounding factors and introduces potential selection bias which affect the interpretation of the results, the study provides valuable real-world insights into the outcomes of PC using the Silo technique. Furthermore, although the pre-operative HbA1c indicated that differences in diabetic control may have affected outcomes, this is limited to the patients’ diabetic control prior to undergoing PC with the Silo technique. Measuring post-operative HbA1c over time could provide more concrete insight into the impact of diabetic control on ulcer healing. During the study period, the hospital transitioned to a new electronic patient record system, resulting in some variability in the completeness of data, particularly for patients treated before the transition. This occasionally limited the ability to identify specific causes of mortality or certain post-operative complications. Additionally, long-term functional outcomes were assessed via telephone follow-up for the 17 surviving patients, conducted at a single time point. The self-report nature of these consultations introduces the risk of recall bias, potentially affecting the outcomes of this study. Although this approach offers a meaningful snapshot of post-operative mobility, future studies with prospective designs and standardised follow-up intervals would allow for a more consistent and comprehensive evaluation of functional recovery over time. Furthermore, the assessment of ambulatory status after surgery was based on clinical documentation from physiotherapy assessments which cannot be relied on with certainty due to the potential for inconsistent documentation and the risk of measurement bias. Future studies would benefit from the use of validated assessment tools such as the LEFS to consistently assess and compare functional outcomes.

The findings of this study align with existing literature on PC for calcaneal osteomyelitis, as well as studies employing local antibiotic delivery devices as adjuncts to surgery. Notably, the five-year mortality rate observed in our cohort is high; however, due to limited data on the specific causes of death, we are unable to draw firm conclusions about the direct impact of PC with the Silo technique on this outcome.

This is only the second study to report outcomes following the Silo technique and our findings reinforce those of the initial report [27]. This case series and its findings should add to the body of literature regarding the use of PC with adjuvant local antibiotic implementation to treat calcaneal diabetic osteomyelitis. The promising outcomes observed here underscore the value of further investigation through prospective cohort studies and randomised controlled trials. Future research comparing PC with the Silo technique and systemic antibiotics against PC with systemic antibiotics alone could help clarify the added benefit of local antibiotic carriers and optimise treatment strategies for this challenging condition.

## Conclusion

PC combined with the Silo technique and adjuvant local antibiotic therapy appears to be an effective surgical option for managing chronic diabetic calcaneal osteomyelitis. This approach demonstrated favourable outcomes in terms of infection eradication, ulcer healing, recurrence rates, and limb salvage, aligning well with existing literature. Functional outcomes were encouraging, with 86.7% of patients maintaining or improving their post-operative ambulatory status. Although there was an overall low one-year all-cause mortality, due to a lack of cause-of-death data this cannot solely be attributed to the Silo technique. While a relatively high five-year all-cause mortality was observed, this likely reflects the complexity and comorbidity burden of the patient population. Due to the retrospective nature and the lack of a control group, the findings from this preliminary study are intended to be exploratory and must be interpreted with caution. Further prospective comparative cohort studies and randomised controlled trials are warranted to better understand the factors influencing long-term outcomes and to evaluate the added value of the Silo technique in comparison to other treatment modalities.

## Supplementary Information

Below is the link to the electronic supplementary material.

Supplementary Material 1

Supplementary Material 2

## Data Availability

No datasets were generated or analysed during the current study.
