# Supplementary material for: Clinical and functional outcomes of the silo technique in the management of diabetic calcaneal osteomyelitis
Source: Cardiovasc Diabetol Endocrinol Rep. 2025 Nov 3;11:26. doi: 10.1186/s40842-025-00238-4 (PMC12581439; doi:10.1186/s40842-025-00238-4)
Supplement: Supplementary file 1 — Supplementary Material 1 [file 40842_2025_238_MOESM1_ESM.docx]

| Microorganism | First debridement | Second debridement | Third debridement |
| --- | --- | --- | --- |
| *Staphylococcus aureus* | 13 | 5 | 2 |
| *Corynebacterium striatum* | 6 | 3 |  |
| *Staphylococcus spp* | 5 |  |  |
| *Candida* | 5 |  |  |
| *Enterococcus spp* | 5 |  |  |
| *Enterobacter cloacae* | 4 |  | 1 |
| *MRSA* | 4 |  | 1 |
| *Streptococcus spp* | 3 | 2 |  |
| *Enterococcus faecalis* | 4 |  |  |
| *Pseudomonas aeruginosa* | 2 | 2 |  |
| *Klebsiella* | 3 |  |  |
| *Escheria coli* | 2 |  |  |
| *Proteus mirabilis* | 2 |  |  |
| *Morganela morgagni* | 1 |  |  |
| *Proteus spp* | 1 |  |  |
| *Peptostreptococcus* | 1 |  |  |

**Table S1.** Tabulated results of each microorganism isolated from the first, second and third debridements.
